# Man and Medicine

**Published:** 1889-01-26

**Authors:** 


### The Hospital

A Weekly Institutional Journal of

Science, Medicine, Nursing, and Philanthropy.

*For Hospitals, Asylums, Medical Practitioners, Students, Nurses, Families, Parishes, Congregations, and the Charitable Public.*

Vol. V. No. 122.] [January 26, 1889.

### Man and Medicine.

If the doctrine of evolution as commonly understood be a truthful presentation of facts and principles, it follows that in order to secure a rational and healthy harmony between science and practice there must be a frequent readjustment of the practical business of the world. It would take us too far afield to discuss the bearing of this proposition in its widest aspects, including, as it does, religion, politics, and all the other leading questions which agitate and influence mankind. At the present time it will be sufficient to approach the domain of medicine, and using evolution as a lighted torch, to turn its blaze upon that part of the established order in which medical science exercises its activity.

Instinct, which is common to man and animals, teaches them to seek relief from pain; conscious intelligence, which is peculiar to man, teaches him to seek that relief intelligently. It seems to be a part of the purposed order of things that high intellectual endowments are to be distributed among several individuals, few or many. All the talents are never concentrated in one person. If a man have more than the average, and have them in a more marked degree, he soon becomes conspicuous among his fellows. In accordance with this law it has happened that some men have been unusually endowed with the faculty of administering to bodily suffering, just as others were endowed with the gift of music, of poetry, of sculpture, or of arithmetic. The original university which conferred degrees upon different men was the University of the World; and the authority which pronounced upon the fitness of the candidates was "common consent."

If it be granted that certain men in all ages have been peculiarly endowed—and it cannot be denied—it is worth while to ask what was the intention of Nature in granting certain endowments to certain persons? Were they so endowed for their own personal advantage, or did Nature, who is neither a fool nor an altogether unkind abstraction, wish to serve some other purpose in their endowment? Did Homer sing for himself? It is probable that if he had had no audience he would never have sung at all. Did Socrates think and teach for himself? If there had been no curious or bewildered men in the world there would have been no record to-day of the life and words of the wise man. It is clear as noonday that in the scheme of things all men do not possess all intellectual gifts; but some men possess one or more pre-eminent faculties, and they are endowed with those faculties in order that they may employ them for the general advantage of mankind. That faculty is the most valuable in itself, and is best used by its possessor which confers the most worthy advantage upon the largest number of persons. The man who considers that a peculiar endowment, whether natural or acquired, is used for the best purpose when it confers the greatest immediate benefit upon himself, is in exactly the same position as that curious class of religious heretics who believe that Almighty God has chosen out a certain number of "elect persons" for immortality, that they themselves are among the fortunate few, and that all the rest will be damned, come what may. This class of persons, who used to be rather numerous, are now so few in number as to be objects of scientific curiosity.

If it be true that the peculiar endowments of certain individuals are conferred upon them not in any sense for their own exclusive advantage, but for the use, well-being, and progress of the race, then any person or body of persons who seek to hoard their intellectual gifts or acquirements for their own exclusive use, or to hinder their general application, must be looked upon as public enemies. The wickedness and culpability of their enmity must be measured by the nature and measure of the faculty they hoard, and by the injury which is inflicted upon the world in consequence. If, for example, the country were in danger from a foreign invader, and a man should have in his possession a simple means of blowing up the enemy's fleet by a single stroke, but should refuse to do it because Parliament was not able to pay him all he demanded for his secret, that man would be an enemy of the basest kind, and his sentence of death would be pronounced in the same breath by the immediate consent of a jury of all his countrymen.

Notwithstanding the self-evident nature of the argument, an argument which nobody but a villain would dispute in the abstract, it has frequently happened in the world's history that faculties and endowments meant for mankind, which mankind needed, and for the want of which mankind has been injured irreparably—such faculties and endowments have been used exclusively for self-advantage, or for the advantage of that larger self which is included under the name of family or of party. As examples of this it is sufficient to mention the action of the three so-called learned professions in times not very remote. Whole periods of history have seen the priesthood exercising the prerogatives of an exclusive caste, shutting up the door of knowledge, and doling out such scraps as from time to time they thought fit to an ignorant and often brutalised people. Similarly the law, at certain epochs, instead of being the highway of justice, was a labyrinth of intricate bye-paths, which seldom led to either justice or right, but only to the enrichment of the legal guides, and to the destruction of those who sought their aid. No less was this the case with medicine, that humanest of all arts. It, too, has often fallen into the hands of cunning and greedy birds of prey, who sometimes did not take the trouble even to acquire knowledge; and who when they had knowledge, wrapped it so round and round with bandages of mystery, that neither they nor their unhappy dupes could gain help or healing. The record of many of the doings of past ages makes every man of open and just mind burn with anger and disgust.

We are fallen to some extent upon better times. But human nature in the mass is not entirely changed. If evolution be in any practical sense true, we are bound to believe that there has been some progression towards a higher intellectuality, and therefore towards a clearer appreciation of the doctrine of a rational altruism. Agnostic philosophers say that high mental culture is the destruction of hard and vulgar self-interest. The evidence of this is not so clear as could be wished, but still it is not altogether wanting. In certain departments of life it is recognised that all knowledge, all experience, and all the stored gains of the ages are for all. Here and there is found a leader of the people whose torch of leadership is held high above the expanding field of universal science, and whose beckoning eye encourages all men to enter and reap.

Assuming the general principles of evolution to be true, where ought we to look for men who will devote themselves to the noble task of making them practically effective? Where, but among the teachers and enthusiastic disciples of the new doctrines? In what profession has evolution found its most numerous and most ardent converts? In the profession of medicine, and especially among those who undertake to teach their fellows by pen and voice. Do these enthusiasts understand the drift and significance of their own doctrines? If in the field we have "first the grain, then the ear, after that the full corn in the ear," so in the world of biological phenomena we have first the protoplasm, then the conscious ego, after that the altruistic consciousness. The altruistic consciousness is held to be the highest product of the evolutionary philosophy, and the church of the evolution finds most of its converts in the ranks of medicine. If this church does not object to the employment of a test found infallible in other churches, we commend it to the consideration of the leaders. "By their fruits ye shall know them" is the unfailing test of the value of all faiths. What fruits of altruism has modern medicine to show? Is it seeking in any urgent, persistent, and intelligent sense the highest material and mental good of the whole community? Or is it to a large extent, using the community solely for its own advantage? If the latter, then medicine is a disgrace to all science, and a scandal to its own peculiar doctrine of evolution. Does any individual medical man consider that we are here ranging in regions beyond what is immediately practical? Then he, at least, has not yet learnt even the alphabet of a genuine evolutionary science. If evolution leads anywhere it leads to altruism in its highest manifestations. If altruism means anything for the medical profession, it means that medicine was made for man, not man for medicine.

